# Effect of hyperbaric oxygen therapy on peripheral blood inflammatory markers in patients with neuromyelitis optica spectrum disorder: a retrospective cohort study

**DOI:** 10.3389/fneur.2025.1670455

**Published:** 2025-11-12

**Authors:** Liya Pan, Yanming Mo, Yulan Wei, Shisheng Luo, Yuan Wu

**Affiliations:** 1 Department of Neurology, The First Affiliated Hospital of Guangxi Medical University, Nanning, China; 2 Department of Hyperbaric Oxygen Therapy, The Fourth Affiliated Hospital of Guangxi Medical University/Liuzhou Workers' Hospital, Liuzhou, China

**Keywords:** hyperbaric oxygen therapy, neuromyelitis optica spectrum disorder, neutrophil-to-lymphocyte ratio, platelet-to-lymphocyte ratio, immunomodulation

## Abstract

**Background:**

Neuromyelitis optica spectrum disorder (NMOSD), an AQP4-IgG-mediated central nervous system demyelinating disease, is prone to recurrent disability. Although the anti-inflammatory and neuroprotective effects of hyperbaric oxygen therapy (HBOT) in neurological diseases have been reported, its immunological impact on NMOSD remains poorly understood.

**Objective:**

To evaluate the effect of HBOT on peripheral inflammatory markers in patients with NMOSD and explore its potential immunomodulatory role.

**Methods:**

This retrospective cohort study included 36 NMOSD patients diagnosed between January 2022 and December 2024, divided into an HBOT plus standard treatment group (*n* = 18) and a standard treatment-only group (*n* = 18). Peripheral blood samples were collected before and after treatment to assess the neutrophil-to-lymphocyte ratio (NLR), platelet-to-lymphocyte ratio (PLR), and lymphocyte counts. Paired tests and ANCOVA (adjusted for baseline values) were used to compare within-group and between-group differences.

**Results:**

After a median of 12 HBOT sessions, the HBOT group showed a 31.78% increase in lymphocyte count (Δ = +0.41 × 10^9^/L, 95% CI: 0.05–0.77), and significant reductions in NLR by 18.98% (Δ = −0.52, 95% CI: −1.02 to −0.02) and PLR by 17.97% (Δ = −37.21, 95% CI: −69.15 to −5.27). After adjustment, the HBOT group demonstrated significantly greater improvements in NLR (−55.56%) and PLR (−22.79%) compared to the control group (both Bonferroni-corrected *P* < 0.01). Subgroup analysis revealed that patients with baseline NLR ≥ 3 benefited the most (interaction *P* = 0.038). No serious adverse events were observed.

**Conclusion:**

HBOT may help rebalance the immune system in NMOSD by increasing lymphocyte counts and reducing NLR and PLR, potentially contributing to immune modulation. These findings support the potential of HBOT as an adjunctive therapy for NMOSD, particularly in patients with a high inflammatory burden. Larger prospective studies are warranted to confirm its long-term efficacy and underlying mechanisms.

## 1 Introduction

Neuromyelitis optica spectrum disorder (NMOSD) is an autoimmune demyelinating disease of the central nervous system, primarily affecting the optic nerves and spinal cord, mediated by aquaporin-4 (AQP4) immunoglobulin G (IgG) antibodies. It can rapidly lead to severe visual and motor disabilities (1). The global annual incidence rate ranges from 0.05 to 0.40 per 100,000 population, with a prevalence of 0.5 to 4 per 100,000. More than 80% of patients are female, reflecting significant gender and racial disparities (2). Although glucocorticoid pulse therapy and long-term immunosuppressive agents (such as rituximab, mycophenolate mofetil, azathioprine, etc.) are currently the mainstay for controlling NMOSD relapses, long-term or high-dose use often leads to side effects such as osteoporosis, infections, and metabolic disorders. Moreover, some patients show poor response to traditional treatment regimens (3, 4). Although targeted biologic agents (such as eculizumab, inilizumab, satralizumab, etc.) launched in recent years can significantly reduce recurrence rates, their high cost and potential infection risks have limited their widespread use (5, 6). Therefore, exploring affordable, risk-controllable, and complementary adjuvant treatment strategies that can improve the long-term prognosis of NMOSD is of significant clinical importance.

Hyperbaric Oxygen Therapy (HBOT) refers to the medical treatment of inhaling 100% oxygen at pressures higher than normal atmospheric pressure (usually 1.5–2.5 ATA) to increase blood oxygen partial pressure, improve tissue oxygenation, and induce a series of cellular protective pathways (7–9). Animal and clinical studies have shown that HBOT can achieve multiple effects, including anti-inflammatory, neuroprotective, and promoting injury repair, by inhibiting reactive oxygen species production, regulating the balance of pro-inflammatory/anti-inflammatory cytokines, suppressing neutrophil extracellular trap (NETs) formation, and improving mitochondrial function (10–13). Furthermore, recent animal and clinical studies have found that HBOT provides new biological evidence for its application in neuroimmune diseases through (1) inhibiting NETs-mediated complement activation, (2) down-regulating Th17/GM-CSF while promoting Treg formation, (3) inducing microglial M1 → M2 polarization, (4) suppressing the NLRP3-pyroptosis pathway, (5) protecting the blood-brain barrier, (6) activating the Nrf2/HO-1 antioxidant axis, (7) closing the NF-κB-chemokine “master switch”, and (8) promoting neurovascular remodeling through multi-target synergy (13–19). In neuro-immune related diseases such as multiple sclerosis, spinal cord injury, and chronic wounds, HBOT has shown potential in reducing neuroinflammation and improving functional outcomes (20, 21). However, its impact on inflammatory response and immune balance in NMOSD patients remains lacking in systematic research.

The neutrophil to lymphocyte ratio (NLR) and platelet to lymphocyte ratio (PLR) are convenient indicators reflecting systemic inflammation and immune homeostasis. Previous studies have shown that higher NLR and PLR levels are closely associated with acute activity, relapse risk, and poor functional prognosis in NMOSD (22–25). Considering HBOT's ability to regulate the inflammatory microenvironment, we speculate that HBOT may exert immunomodulatory effects by improving inflammatory markers such as NLR and PLR, thereby reducing systemic inflammatory response in NMOSD patients. This provides insights into exploring the “hemogram-inflammation-prognosis” chain and offers new adjuvant treatment options for NMOSD.

Based on this, the present study adopted a retrospective cohort design to compare the effects of hyperbaric oxygen therapy (HBOT) combined with standard treatment vs. standard treatment alone on peripheral blood neutrophil-to-lymphocyte ratio (NLR), platelet-to-lymphocyte ratio (PLR), and lymphocyte counts in patients with neuromyelitis optica spectrum disorder (NMOSD), aiming to elucidate the immunological effects of HBOT and preliminarily assess its clinical feasibility, thereby providing evidence for subsequent randomized controlled trials.

## 2 Materials and methods

### 2.1 Research design

This study is a single-center, retrospective cohort study that included patients who met the diagnostic criteria for neuromyelitis optica spectrum disorder (NMOSD) between January 2022 and December 2024. All patients had been receiving stable immunosuppressive therapy for at least 8 weeks and had not experienced any acute attacks before enrollment. The disease duration ranged from 1 month to 5 years.

### 2.2 Research object

#### 2.1.1 Inclusion criteria

Meets the 2015 international diagnostic criteria for NMOSD. The duration of disease since first diagnosis is ≥1 month and ≤ 5 years. In remission: No relapses have occurred in the past 3 months. Stable treatment: Patients must have received a stable dose of immunosuppressants (such as rituximab, azathioprine, or mycophenolate mofetil) for at least 8 consecutive weeks before enrollment, and may be combined with oral low-dose prednisone (≤ 20 mg/day).Patients aged between 18 and 65 years, regardless of sex, who had signed the informed consent form and were able to cooperate with follow-up visits.

#### 2.1.2 Immunosuppressive agent usage

In this study, patients received immunosuppressive agents including azathioprine (AZA), mycophenolate mofetil (MMF), and rituximab (RTX). These medications have different mechanisms of action but are all used to suppress immune responses and reduce disease relapses in NMOSD patients. Before the study commencement, the immunosuppressive treatment regimens for all patients were determined by neurologists based on each patient's clinical history and disease needs, ensuring that each patient maintained stable immunosuppressive therapy for at least 8 weeks before enrollment.

- AZA: By inhibiting T cell proliferation, reducing the immune system's attack on neural tissue, it is suitable for long-term immunosuppressive therapy.
- MMF: By suppressing the activity of B cells and T cells to reduce immune response, it is particularly suitable for long-term immunotherapy.
- RTX: By targeting and eliminating B cells to suppress specific immune responses, this approach is typically used in cases with strong drug resistance (Table 1 shows the frequency of use for each immunosuppressive agent in both groups.)

**Table 1:** Baseline characteristics of patients and between-group comparisons.

**Variable**	**Observation group (HBOT + standard treatment, *n =* 18)**	**Control group (standard treatment only, *n =* 18)**	***P*-value**
Age (years)	45.67 ± 12.30	46.38 ± 13.18	0.871 (*t*-test)
Female (%)	15 (83.33%)	15 (83.33%)	1.000 (Fisher)
BMI (kg/m^2^)	23.8 ± 3.2	24.1 ± 3.4	0.78
Disease duration (months)	22 (10–38)	24 (11–36)	0.64
Cumulative relapse count	2 (1–4)	2 (1–5)	0.71
Hypertension, *n* (%)	4 (22.2%)	3 (16.7%)	0.67
Diabetes, *n* (%)	2 (11.1%)	1 (5.6%)	0.55
AQP4-IgG Positive, *n* (%)	15 (83.3 %)	16 (88.9 %)	1.000
Baseline NLR, M (P25–P75)	2.74 (2.03–8.81)	3.73 (1.95–5.83)	0.401 (U-test)
Baseline PLR, M (P25–P75)	207.04 (163.50–298.20)	164.69 (132.40–249.60)	0.102 (U-test)
CRP (mg/L), M (P25–P75)	0.75 (0.50-2.50)	0.55 (0.28–1.20)	0.181 (U-test)
Body temperature (°C), Mean ± SD	36.7 ± 0.30	36.8 ± 0.40	0.45 (*t*-test)
Chest imaging negative, *n* (%)	18 (100 %)	18 (100 %)	1.000
Prednisone dose (mg/day), M (P25–P75)	10.00 (5.00–15.00)	12.50 (5.00–20.00)	0.312 (U-test)
Immunosuppressant use, *n* (%)	12 (66.70%)	14 (77.80%)	0.714 (Fisher)
Immunosuppressant types, *n* (%)	AZA:4 (22.2%)	AZA: 5(27.8%)	0.70
	MMF: 5 (27.8%)	MMF: 6 (33.3%)	0.80
	RTX: 3(16.7%)	RTX: 3 (16.7%)	1.00

1. Age, BMI, disease duration: Continuous variables are presented as mean ± standard deviation (SD), and comparisons between groups were made using an independent t-test. 2. Cumulative relapse count: Data presented as median (P25–P75), and between-group comparisons were made using the Mann–Whitney U-test. 3. Hypertension and diabetes: Categorical variables are presented as n (%) and were compared using Fisher's exact test. 4. AQP4-IgG positive: Percentage of patients with positive AQP4-IgG, compared between groups using Fisher's exact test. AQP4-IgG was detected by cell-based assay (CBA), with a titer ≥1:10 considered positive. 5. NLR, PLR, and CRP: Neutrophil-to-lymphocyte ratio (NLR), platelet-to-lymphocyte ratio (PLR), and C-reactive protein (CRP) are presented as median (P25–P75), and comparisons were made using the Mann–Whitney U-test. 6. Chest imaging negative: All patients had negative chest imaging results, and between-group comparisons showed no significant difference. 7. Immunosuppressant use: Immunosuppressants included rituximab, azathioprine, and mycophenolate mofetil. Data for immunosuppressant use are presented as n (%) and compared using Fisher's exact test. 8. Prednisone dose: Prednisone dose (mg/day) is presented as median (P25–P75), with comparisons made using the Mann–Whitney U-test. 9. Note: For all indicators at the 4th week, data were consistent with baseline values, with Δ = 0 (0%). Immunosuppressive agents included rituximab, azathioprine, and mycophenolate mofetil; the corticosteroid dose was equivalent to prednisone.

#### 2.1.2 Exclusion criteria

Acute-phase NMOSD, defined as relapse or IVMP pulse therapy (>1 g/day for 3–5 days) within the past 3 months; Recent intensive immunotherapy, including plasma exchange, intravenous immunoglobulin (IVIG), or other biological agents (except maintenance rituximab) within the past 3 months; Active infection within 7 days before enrollment, evidenced by body temperature >37.3 °C, C-reactive protein (CRP) >10 mg/L, or abnormal chest imaging; Concomitant autoimmune diseases (e.g., systemic lupus erythematosus, Sjögren's syndrome);Concomitant major central nervous system diseases (e.g., multiple sclerosis, ischemic stroke, brain tumor);Concomitant severe systemic diseases (e.g., active tuberculosis, malignancy, severe hepatic or renal insufficiency, congestive heart failure);Contraindications to hyperbaric oxygen therapy (e.g., uncontrolled pneumothorax, acute otitis media, recent ear/nasal surgery, or inability to tolerate pressurization);Pregnancy or breastfeeding; Missing essential follow-up data, or any other condition judged by the investigators to render the patient unsuitable for participation.

### 2.3 Intervention measures

#### 2.3.1 HBOT protocol

The treatment was administered using the GY3800M6-C medical hyperbaric oxygen chamber (2.0 ATA, certified by the National Medical Products Administration) once daily. The compression phase was conducted at a rate of 4–20 kPa/min, starting slowly and then increasing, with a total compression time of 20 min to reach the target pressure of 2.0 ATA. The stabilization phase lasted 65 min, during which patients received oxygen therapy for a total of 60–30 min of oxygen inhalation followed by a 5-min break, then another 30 min of oxygen inhalation. The decompression phase took 20 min at a rate of 4–20 kPa/min, starting quickly and then slowing down. The entire session lasted 105 min, with 12 sessions constituting one course of treatment. Patients who completed ≥5 sessions were included in the analysis. Throughout the procedure, certified technicians monitored the chamber's oxygen concentration and the patients' vital signs.

#### 2.3.2 Prednisone dosage adjustment and control

As an immunosuppressive drug, prednisone is known to affect the counts of lymphocytes and neutrophils, which may alter the neutrophil-to-lymphocyte ratio (NLR). To control for the potential impact of prednisone dosage on immune markers, this study employed the following statistical methods.

##### 2.3.2.1 The prednisone dose was adjusted as a covariate

In all statistical analyses, we used analysis of covariance (ANCOVA) to adjust for baseline prednisone dosage as a covariate. The use of prednisone may lead to lymphopenia and neutropenia; therefore, we employed this method to reduce the potential interference of prednisone on NLR and PLR, ensuring that our evaluation results reflect the independent effects of hyperbaric oxygen therapy.

##### 2.3.2.2 Sensitivity analysis

To further verify the impact of prednisone dosage on outcomes, we conducted a sensitivity analysis, excluding patients whose prednisone dosage was adjusted by more than 5 mg/day during treatment. All patients in the study were receiving a dose of prednisone ≤ 20 mg/day. Through this measure, we ensured that fluctuations in prednisone dosage would not significantly affect changes in NLR and PLR, thereby guaranteeing the robustness of statistical results.

#### 2.3.3 Standard treatment

In this study, all patients received standard treatment, including immunosuppressive agents and glucocorticoids (prednisone ≤ 20 mg/day). It is important to note that this regimen of combined immunosuppressants with low-dose prednisone as maintenance therapy reflects common clinical practice in China for managing NMOSD in remission. However, it is noteworthy that the use of long-term low-dose steroids in stable NMOSD is not a universal standard and may not be applicable in all regions. The immunosuppressive agents used included azathioprine (AZA), mycophenolate mofetil (MMF), and rituximab (RTX), which were selected based on each patient's clinical history and treatment response. All treatment regimens and dosage adjustments were determined by neurologists according to clinical guidelines.

To ensure immune system stability and minimize external interference, all patients were required to have stable immunosuppressive therapy for at least 8 weeks before enrollment and initiation of hyperbaric oxygen therapy. During follow-up, if tapering was required, prednisone dose reduction was limited to ≤ 2.5 mg every 2 weeks, and no new immunomodulatory drugs were permitted. As such, the low-dose prednisone (≤ 20 mg/day) in this study was strictly employed as a maintenance therapy to stabilize immune status during remission, and is distinct from high-dose corticosteroid pulse therapy used for acute relapse.

#### 2.3.4 Concurrent treatment control

In this study, all patients continued their standard treatment protocols while receiving hyperbaric oxygen therapy to ensure effective immune system suppression without excessive immune response. In addition to standard immunosuppressive therapy, patients were not permitted to add new immunomodulatory drugs during the treatment period. Throughout the treatment, prednisone dosage adjustments and control followed standard operating procedures to ensure consistency in immunosuppressive treatment for all patients during hyperbaric oxygen therapy.

### 2.4 Data collection and indicator measurement

#### 2.4.1 Hematological parameters

Fasting venous blood samples were collected between 08:00 and 09:00, and tested 24 h before the first HBOT session (baseline) and 24 h after the last treatment. The Expanded Disability Status Scale (EDSS) was assessed at these same timepoints: at baseline and within 24 h after the final treatment session (i.e., at the end of the 4-week intervention period). Neutrophils (NEU), lymphocytes (LYM), platelets (PLT), etc., were measured using the Beckman DxH-900 fully automated analyzer, and NLR (NEU/LYM), PLR (PLT/LYM), and MLR (MONO/LYM) were calculated.

#### 2.4.2 High inflammation load threshold setting

In this study, NLR = 3 was used as the threshold for high inflammatory load, based on the following considerations: In healthy populations, NLR ≈3 serves as the reference upper limit, and any value exceeding this suggests systemic inflammation activation; Multiple NMOSD studies have shown that NLR ≥3 is significantly associated with short-to-medium-term relapses and disability progression, and has been recommended for risk stratification; Compared with thresholds such as 2.5 or 4.0, a threshold of 3 achieves a balance between sensitivity and specificity, while being clinically easy to operate; The median baseline NLR in this cohort is 2.74, and a cutoff value of 3 can highlight the characteristics of high-risk populations while maintaining statistical power (23–28).

#### 2.4.3 Covariates

Age, gender, disease duration, AQP4-IgG status, type of immunosuppressant, and prednisone dosage.

#### 2.4.4 Quality control

Blood samples were analyzed by blinded laboratory personnel, and EDSS scores were independently assessed by two certified neurologists who were also blinded to the study groups.

### 2.5 Statistical analysis

#### 2.5.1 Sample size calculation

Based on previous interventional studies on inflammatory markers in NMOSD (9, 13, 22, 24), the standardized effect size (Cohen's *d*) for the primary endpoint NLR was preset at 0.80 (α = 0.05, β = 0.20, two-tailed test). Using G^*^Power 3.1 software, the minimum sample size per group was calculated as 16 cases. Considering a potential 20% data loss rate (such as incomplete follow-up or missing indicators), 18 patients were finally included in each group, resulting in a total sample size of 36 cases, to ensure sufficient statistical power for the study.

#### 2.5.2 Main analysis

Between-group comparisons were conducted using analysis of covariance (ANCOVA), adjusting for baseline NLR, PLR, lymphocyte count, and prednisone dosage. An analysis of covariance (ANCOVA) was employed to minimize potential interference from immunosuppressant use. The type of immunosuppressant was included as a covariate for adjustment purposes. This adjustment aimed to eliminate potential bias that different immunosuppressants might introduce into the study results, ensuring that our assessments reflect the independent effect of hyperbaric oxygen therapy and thereby enhancing the accuracy and reliability of the findings. Using analysis of covariance (ANCOVA) similarly, we reduced the interference of prednisone dosage on immune cell counts and immune marker changes, and adjusted baseline prednisone dosage as a covariate. Through this method, we could control the potential impact of prednisone dosage on immune cell counts and immune markers (such as NLR and PLR), thereby ensuring accurate assessment of the independent effects of hyperbaric oxygen therapy on the immune system; within-group pre-post comparisons were performed using a paired *t*-test (for normal distribution) or Wilcoxon signed-rank test (for non-normal distribution).

#### 2.5.3 Effect size and correction

Continuous variables are reported as mean differences (Δ) with 95% confidence intervals (CI), while categorical variables are presented as frequencies (%). The primary outcomes (NLR, PLR) were adjusted by Bonferroni correction (significance threshold *P* < 0.025), with a threshold of *P* < 0.05 for secondary outcomes. Sensitivity analysis: cases with hormone dose changes >5 mg/d during treatment were excluded to verify the robustness of the results.

#### 2.5.4 Software

SPSS 26.0 (IBM); sample size calculated using G^*^Power 3.1 (Cohen's *d* = 0.80, α = 0.05, β = 0.20).

### 2.6 Ethical statement

This study was approved by the Ethics Committee of Liuzhou Workers' Hospital (Approval No. KY2025596). Due to the retrospective design and anonymized data, informed consent was waived.

## 3 Result

### 3.1 Baseline characteristics

A total of 36 NMOSD patients were enrolled (18 in the HBO + standard treatment group and 18 in the control group). There were no significant differences between the two groups in terms of age (45.67 ± 12.30 vs. 46.38 ± 13.18 years), gender (female 83.33% vs. 83.33%), immunosuppressant usage rate, prednisone dosage, and baseline NLR/PLR (all *P* > 0.05), suggesting good comparability (Table 1). Among the 36 patients, 31 (86.1%) were AQP4-IgG positive and 5 (13.9%) were AQP4-IgG negative, with the latter divided between the treatment group (3 patients) and the control group (2 patients).

### 3.2 Between-group comparison of major inflammation indicators (after ANCOVA adjustment)

After 4 weeks of treatment, the adjusted mean NLR in the hyperbaric oxygen group was significantly lower than that in the control group (2.20 vs. 4.95, Δ = −2.75, partial η^2^ = 0.34, 95% CI −4.11 to −1.39, Bonferroni-corrected *P* = 0.001), and the PLR was also significantly reduced (170.10 vs. 220.30, Δ = −50.20, partial η^2^ = 0.29, 95% CI −83.40 to −17.00, corrected *P* = 0.003). Meanwhile, lymphocyte count increased (1.68 vs. 1.52 × 10^9^/L, Δ = +0.16, partial η^2^ = 0.17, 95% CI +0.04 to +0.28, corrected *P* = 0.012) (Table 2). To account for potential baseline imbalances, an analysis of covariance (ANCOVA) was performed, adjusting for baseline NLR, PLR, lymphocyte count, and prednisone dosage. After adjustment, the HBOT group remained significantly superior to the control group in reducing NLR (adjusted *P* = 0.041) and PLR (adjusted *P* = 0.032), and in increasing lymphocyte count (adjusted *P* = 0.027), indicating that the observed effects were independent of baseline differences.

**Table 2:** Comparison of inflammation markers between groups (ANCOVA, adjusted for baseline).

**Index**	**Observation group adjusted mean**	**Control group adjusted mean**	**Inter-group Δ**	**Δ%**	**95 %CI**	**Partial η^2^**	**Inter-group *P-*value**	**Inter-group *P*-value**
NLR	2.20	4.95	−2.75	−55.56 %	−4.11 to −1.39	0.34	0.038^*^	0.001^**^
PLR	170.10	220.30	−50.20	−22.79 %	−83.40 to −17.00	0.29	0.048^*^	0.003^**^
Lymphocyte (× 10^9^/L)	1.68	1.52	+0.16	+10.53 %	+0.04 to +0.28	0.17	0.041^*^	0.012^*^

Δ = Observed group – Control group adjusted mean difference; Partial η^2^ represents effect size; Raw P = ANCOVA result

Comparisons for primary outcomes (NLR, PLR) use Bonferroni correction, threshold = 0.025; ^*^P < 0.05; ^**^P < 0.025.

### 3.3 Within-group changes

#### 3.3.1 Observation group

**Figure 1:**
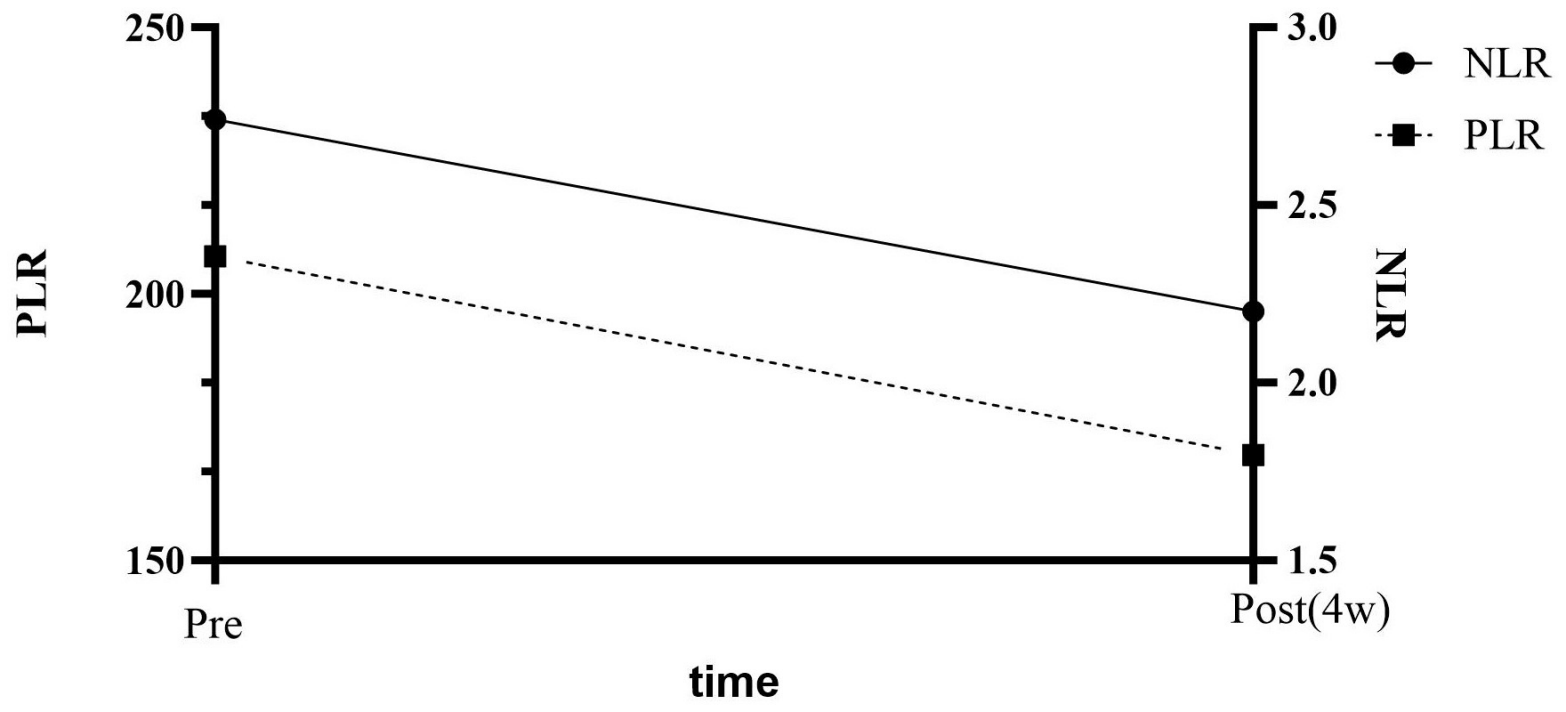
Dynamic changes of NLR and PLR before and after hyperbaric oxygen therapy.

WBC increased from 8.19 ± 3.79 to 9.79 ± 3.52 × 10^9^/L (Δ +1.60, Δ% +19.54%, *d* = 0.43, *P* = 0.032; 95% CI 0.15–3.05); the median NLR decreased from 2.74 to 2.22 (Δ −0.52, δ = 0.35, *P* = 0.043); PLR decreased from 207.04 to 169.83 (Δ −37.21, δ = 0.42, *P* = 0.025); lymphocyte count increased to 1.70 × 10^9^/L (Δ +0.41, δ = 0.38, *P* = 0.028) (Table 3, Figure 1).

**Table 3:** Intra-group comparison of blood cells and inflammation indicators in the observation group (*n* = 18).

**Index**	**Pre-treatment**	**Post-treatment**	**Δ**	**Δ%**	**95 %CI**	***P-*value**	**Effect size (d/δ)**	**FDR corrected *P***
WBC (× 10^9^/L)	8.19 ± 3.79	9.79 ± 3.52	+1.60	+19.54 %	+0.15 to +3.05	0.032^*^	*d =* 0.43	0.096
NLR	2.74 (2.03–8.81)	2.22 (1.87–6.94)	−0.52	−18.98 %	−1.02 to −0.02	0.043^*^	δ = 0.35	0.129
PLR	207.04 (163.50–298.20)	169.83 (85.00–211.00)	−37.21	−17.97 %	−69.15 to −5.27	0.025^*^	δ = 0.42	0.100
Lymphocyte (× 10^9^/L)	1.29 (0.95–1.84)	1.70 (1.13–1.73)	+0.41	+31.78 %	+0.05 to +0.77	0.028^*^	δ = 0.38	0.112

Δ = Post-Treatment – Baseline; d = Cohen d (for normal variables), δ = non-parametric effect size for paired data; FDR P is corrected using the Benjamini–Hochberg method. ^*^P < 0.05.

#### 3.3.2 Control group

Only WBC and monocyte counts showed mild elevations (*P* < 0.05), while NLR, PLR, and lymphocyte counts remained unchanged.

### 3.4 Subgroup analysis and sensitivity analysis

#### 3.4.1 Subgroup analysis

**Figure 2:**
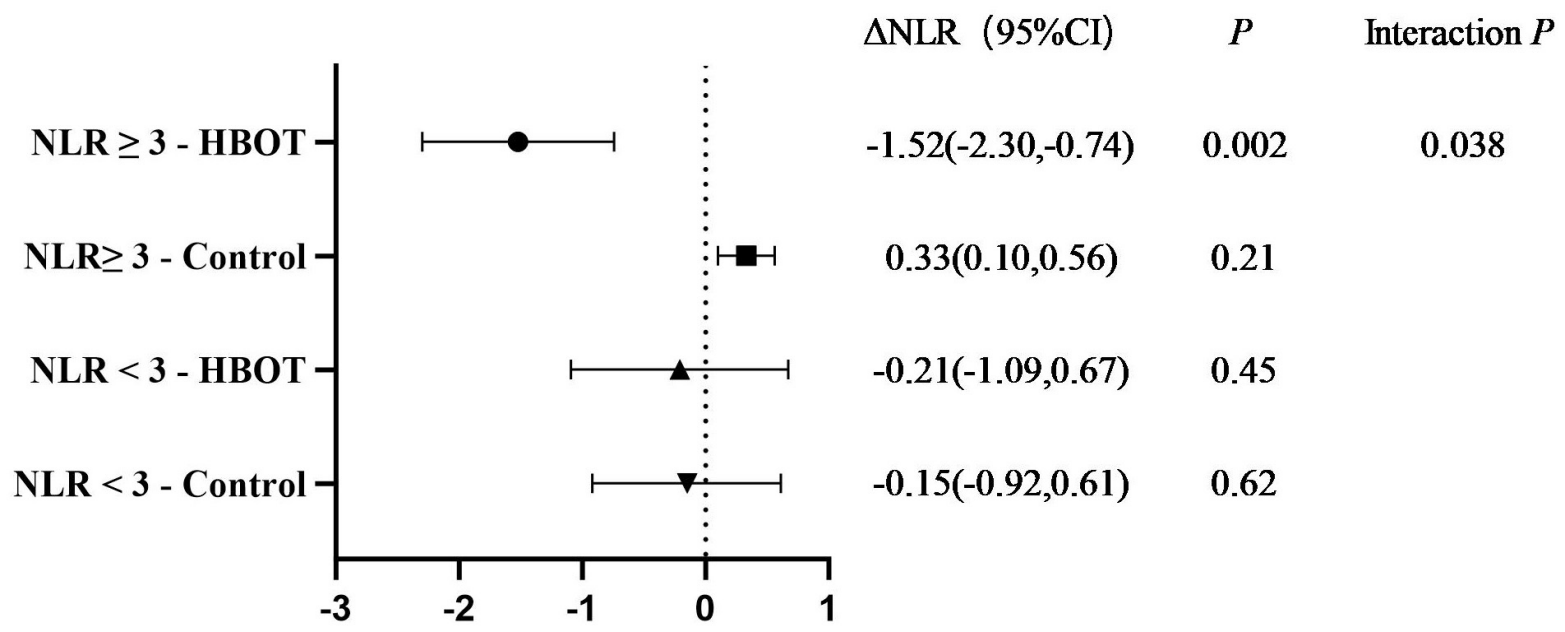
Comparison of NLR changes between the HBOT group and control group after treatment in different baseline NLR subgroups. NLR = 3 is the critical value for high inflammatory load, and the rationale can be found in the high inflammatory load threshold setting.

In the subgroup with baseline NLR ≥ 3 (*n* = 20), the hyperbaric oxygen group showed a significant decrease in NLR by −1.52 (95% CI −2.30 to −0.74), while the control group showed no significant change (+0.33); the interaction *P* = 0.038 suggested greater benefit in patients with higher inflammatory burden (Figure 2). Subgroup analysis based on baseline inflammatory load revealed that in patients with a baseline NLR ≥ 3 (*n* = 20), the HBOT group showed a significant reduction in NLR compared to the control group (interaction *P* = 0.038). In contrast, among patients with a baseline NLR <3 (which included participants from both the HBOT and control groups), no significant treatment effect of HBOT on NLR was observed. This suggests that the benefit of HBOT was more prominent in patients with a higher pre-treatment inflammatory burden.

#### 3.4.2 Sensitivity analysis

After excluding two extreme values, the PLR result remained significant (adjusted *P* = 0.028), while the significance of WBC decreased to the marginal level (adjusted *P* = 0.051), indicating the robustness of the primary outcomes (NLR, PLR) (Table 4).

**Table 4:** Sensitivity analysis of observation group (after removing outliers).

**Index**	**Original *P*-value (*n =* 18)**	***P*-value after removing outliers (*n =* 16)**	**Conclusion**
WBC(× 10^9^/L)	0.032^*^	0.051	Marginal results
PLR	0.025^*^	0.028^*^	Robust results

^*^P < 0.05.

### 3.5 Safety and functional outcome

Two patients (11.1%) experienced transient ear fullness during HBOT, but no barotrauma or other serious adverse events occurred. As shown in Table 5, changes in EDSS scores from baseline to the 4-week post-treatment assessment were minimal and not statistically significant within either group (observation group: 3.5 → 3.3, *P* = 0.150; control group: 3.6 → 3.5, *P* = 0.210), with no significant difference between the groups (*P* = 0.620). This indicates that a significant short-term improvement in EDSS was not observed within the 4-week study period.

**Table 5:** Changes in EDSS scores before and after treatment.

**Index**	**Group**	**Pre-treatment**	**Post-treatment**	***P*-value (within group)**	**Between-group *P-*value**
EDSS Score	Observation	3.5 (2.0–4.0)	3.3 (2.0–4.0)	0.150	0.620
	Control	3.6 (2.5–4.5)	3.5 (2.0–4.5)	0.210	

## 4 Discussion

### 4.1 Research background and significance of preliminary findings

This retrospective cohort study included 36 patients with NMOSD. After 12 sessions of HBOT, the median NLR decreased from 2.74 to 2.22 (Δ = −0.52, *P* = 0.043), and the median PLR decreased from 207.04 to 169.83 (Δ = −37.21, *P* = 0.025) (Figure 1). The simultaneous decrease in NLR/PLR suggests that HBOT produced a systemic anti-inflammatory effect within 4 weeks. Lymphocyte count increased by 0.41 × 10^9^/L (*P* = 0.028). Patients with baseline NLR ≥ 3 showed a greater reduction (Δ NLR = −1.52, interaction *P* = 0.038), suggesting that HBOT has a more significant risk-reducing effect on populations with high inflammatory burden. This study first investigated the effects of HBOT on peripheral blood inflammation markers in NMOSD patients. The results showed that HBOT treatment significantly reduced NLR and PLR while significantly increasing lymphocyte count, suggesting its potential anti-inflammatory effect through regulating immune cell balance. This finding provides preliminary evidence for HBOT as an adjuvant therapy for NMOSD, particularly in patients with baseline NLR ≥ 3, where the effect of HBOT was more significant (ΔNLR = −1.52, interaction *P* = 0.038). These results provide a theoretical foundation for clinical practice and suggest novel avenues for future research. Moreover, although this study did not specifically analyze the timing of HBOT initiation or disease duration, our findings suggest that patients with higher baseline NLR responded more significantly to HBOT. This implies that treatment efficacy may be associated with the degree of systemic immune activation rather than solely with disease stage. Future studies should consider incorporating disease duration and treatment onset timing as variables to further clarify their influence on therapeutic outcomes.

### 4.2 Immunomodulatory mechanisms of hyperbaric oxygen therapy

Previous studies have shown that HBOT achieves immune regulation through multiple mechanisms, including improving tissue oxygenation, inhibiting reactive oxygen species (ROS) production, regulating the balance of pro-inflammatory and anti-inflammatory cytokines, and enhancing mitochondrial function (10–13). The results of this study indicate that HBOT can significantly reduce NLR and PLR while increasing lymphocyte count, which may be associated with decreased activation levels of neutrophils and platelets, as well as improved lymphocyte function. Furthermore, HBOT also suppresses excessive inflammatory responses by improving the function of immune cells, a mechanism closely related to the optimization of the immune microenvironment in NMOSD patients.

#### 4.2.1 The potential regulatory mechanism of HBOT on AQP4-IgG-mediated immune response

**Figure 3:**
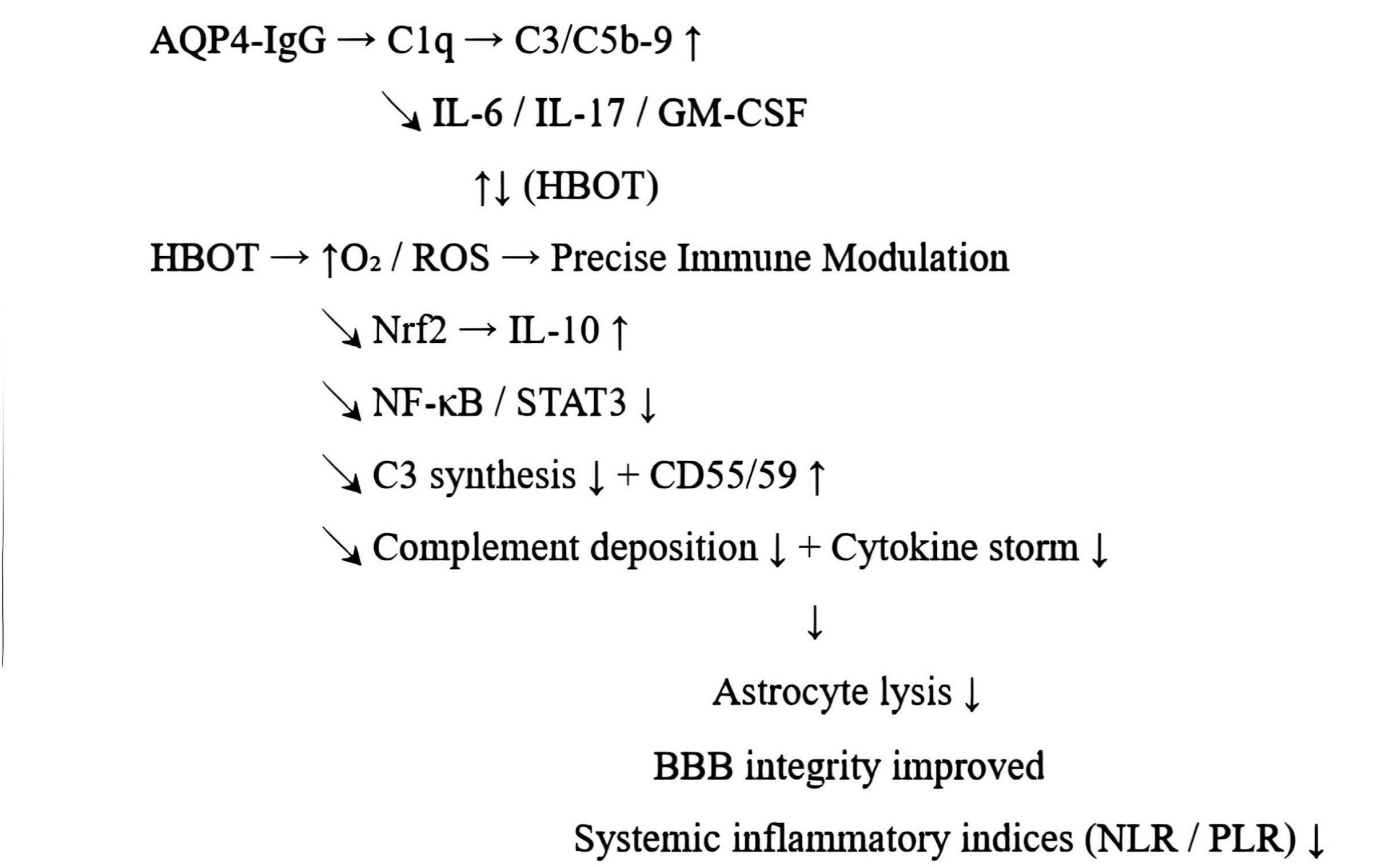
Schematic diagram of HBOT's regulatory mechanism on AQP4-IgG-mediated inflammatory cascade in NMOSD.

In NMOSD, anti-AQP4-IgG binds to astrocyte membranes and rapidly activates the classical complement pathway (C1q → C4/2 → C3 → C5b-9). The released C3a/C5a recruits neutrophils and eosinophils, and MAC formation at focal blood-brain barrier sites directly leads to astrocyte lysis. Meanwhile, pro-inflammatory cytokines such as IL-6, IL-17, GM-CSF, and TNF-α maintain plasma cell survival, enhance BBB permeability, and exacerbate tissue damage. The resulting “complement-cytokine coupling loop” is the core of AQP4-IgG pathogenicity (Figure 3).

#### 4.2.2 HBOT may intervene at the key nodes of this coupling loop.

##### 4.2.2.1 Inhibit complement cascade (C3↓/C5b-9↓)

The hyperoxic environment reduces ischemia-reperfusion ROS peak and inhibits NF-κB/HIF-1α-driven complement synthesis. Renal ischemia-reperfusion models suggest that HBOT (2 ATA) within 3–5 h significantly downregulates ICAM-1, VCAM-1, C3 mRNA and protein levels, and reduces tissue complement deposition (29). HBOT enhances the activity of complement regulatory molecules (CD55/CD59) expressed by astrocytes. Human evidence suggests that serum C3 levels decrease by ≈27% after 10 HBOT sessions in patients with severe atopic dermatitis (30).

##### 4.2.2.2 Rapid down-regulation of core pro-inflammatory cytokines

Continuous hyperbaric oxygen → mitochondrial repolarization → inhibition of NLRP3 and STAT3/RORγt axis, resulting in reduced IL-6/IL-17 levels. There were 242 cases of NSTI patients who underwent paired testing before and after each HBOT session: the median decrease of IL-6 was 7–30 pg mL^−1^ per session, with synchronous decrease in G-CSF (31). ROS fluctuation (“oxygen swing”) induces Nrf2 and HO-1, indirectly increasing IL-10. In the EAE model, semi-therapeutic HBOT inhibits Th17/GM-CSF and up-regulates IL-10, significantly reducing demyelination (13).

##### 4.2.2.3 Remodeling the peripheral immune profile

Retrospective NMOSD cohort (*n* = 36): After 12 sessions of HBOT, lymphocytes increased by 31.78%, NLR decreased by 18.98%, PLR decreased by 17.97%, and patients with high baseline NLR showed the greatest benefit. Improved tissue oxygenation → reduced stress-induced granulocyte mobilization; may shift Treg/Breg metabolism from glycolysis to oxidative phosphorylation, enhancing their suppressive activity.

##### 4.2.2.4 Protect BBB and astrocytes

Multiple CNS injury models have shown that HBOT alleviates edema, stabilizes tight junction proteins, and reduces barrier disruption mediated by C5b-9 and MMP-9. Hyperbaric oxygen elevates vascular endothelial NO levels simultaneously, restoring pericyte function; down-regulation of C3/C5a reduces neutrophil/eosinophil degranulation, thereby alleviating secondary injury.

Based on previous experimental data showing that HBOT inhibits NETs-complement activation and promotes Treg, and considering the findings of this study, it can be reasonably speculated that HBOT attenuates AQP4-IgG-mediated cascade inflammation by rapidly down-regulating NLR/PLR, ultimately reducing systemic inflammatory burden.

### 4.3 The regulatory effect of hyperbaric oxygen therapy on inflammatory markers

This study found that HBOT significantly reduced NLR and PLR, while having no significant direct impact on neutrophil and monocyte counts. This suggests that HBOT may exert its immunomodulatory effects through functional regulation rather than simply altering cell numbers. This finding is consistent with studies in neurological diseases such as multiple sclerosis. The immunomodulatory effects of HBOT may be achieved through multiple mechanisms, such as enhancing anti-inflammatory responses and suppressing excessive inflammatory reactions by regulating the activity of T cells and B cells (32). Furthermore, HBOT has demonstrated potential neuroprotective effects in improving inflammatory responses in NMOSD patients, which may contribute to the repair of demyelinating lesions (33, 34).

### 4.4 The role of NLR and PLR in inflammatory response

As common inflammatory markers, NLR and PLR are widely used in the study of immune-related diseases. Research has shown that elevated NLR and PLR levels are closely associated with acute attacks, relapse risk, and immune imbalance in NMOSD. Multiple studies have consistently shown that when NLR ≥ 3.0, it predicts short-term relapse and higher disability risk; MRI/AQP4 titer re-examination is recommended within 1 month when exceeding the threshold. PLR ≥ 195–200 is significantly correlated with EDSS worsening and BBB disruption within 6 months to 2 years (22, 23, 35–37). During the acute phase of NMOSD, neutrophil and platelet activation participate in the AQP4 antibody-dependent complement cascade reaction, while transient lymphocyte depletion leads to increased NLR and PLR (25). Unlike studies conducted during the acute phase, this study focused exclusively on patients in the remission phase. Therefore, the conclusions drawn here may not be directly applicable to patients in the acute phase of NMOSD. This study further validates the effectiveness of HBOT in regulating these inflammatory markers. Particularly in patients with higher baseline NLR, the improvement in NLR after HBOT treatment was more significant, supporting the theoretical and evidence-based basis of NLR and PLR as early detection indicators (34). This is consistent with the rapid down-regulation of NLR/PLR observed in HBOT studies on diabetic foot and carbon monoxide poisoning (32, 33). The latest multicenter acute NMOSD cohort study (*n* = 154) confirmed that baseline PLR >195 can predict EDSS deterioration within 3 months (AUC = 0.78) (38), suggesting that PLR can serve as a predictor of short-term functional outcomes.

In NMOSD, NLR/PLR is not only a surrogate marker for “current inflammatory burden,” but has been repeatedly confirmed to be associated with future relapse risk, rate of functional deterioration, and increased EDSS scores. Existing evidence-based studies suggest that high NLR/PLR indicates poorer long-term outcomes (24). Elevated NLR at first presentation significantly increases the risk of recurrence (HR = 1.7), with recurrence occurring within 12 months. PLR > 195 independently predicts EDSS worsening (AUC = 0.78), with EDSS deterioration occurring within 3–6 months (38). PLR is the only independent predictor of EDSS ≥ 4 and cross-sectional disability level (22).NLR is positively correlated with cumulative disability and cognitive impairment, leading to long-term functional/cognitive impairment (39). Since NLR ≥ 3 has been regarded as the critical point of poor prognosis in NMOSD, the long-term benefits suggested by this study showed the greatest reduction in the high-inflammation subgroup (baseline NLR ≥ 3) with ΔNLR = −1.52; the group × subgroup interaction *P* = 0.038, suggesting that the addition of HBOT was associated with the most substantial ‘de-risking' effect on high-risk populations, as measured by the reduction in NLR. The NLR decreased by 55.56% and the PLR decreased by 22.79%, reaching significant levels within 4 weeks. Such a magnitude of biomarker reduction has been historically associated with 6–12 months of EDSS stability or improvement in previous NMOSD immunosuppressive or biologic therapy studies (40). Biological rationality: HBOT inhibits neutrophil activation, improves lymphocyte exhaustion → reduces complement-mediated AQP4 damage cascade; meanwhile, it enhances oxygenation and alleviates tissue ischemia-reperfusion response, contributing to long-term axonal preservation.

### 4.5 The synergistic potential of hyperbaric oxygen therapy with existing NMOSD treatments

The conventional management of NMOSD involves long-term immunosuppressants to prevent relapses, coupled with short-term, high-dose glucocorticoids to treat acute attacks (3). It is important to note that in this study, all enrolled patients were in a remission phase and were only receiving low-dose prednisone (≤ 20 mg/day) as part of their stable maintenance regimen, not high-dose acute therapy. As a non-immunosuppressive treatment, HBOT has shown unique advantages in immune regulation. Research indicates that HBOT may become an effective adjunct therapy for NMOSD by improving immune tolerance and reducing the side effects of immunosuppressive treatments, particularly showing greater potential in patients with higher immune burden.

### 4.6 Safety/tolerability

The treatment was well-tolerated, with only two instances (11.1%) of transient ear fullness reported, and no cases of barotrauma or other serious adverse events. Previous studies have shown that ear discomfort/otitis media barotrauma is the most common adverse reaction to HBOT: a systematic review of randomized controlled trials reported an incidence of ear discomfort of approximately 7.5% (113/1,497 cases) (41). Large retrospective studies and reviews have shown an overall range of 8%−68.7% (42), with an average of approximately 13% for commonly used 2.0–2.5 ATA protocols (43). The 11.1% incidence rate of this study falls within the lower-middle range of the aforementioned interval, and all cases were mild and reversible reactions, indicating good tolerability of this regimen. Our observed incidence of aural fullness (11.1%) was lower than or comparable to commonly reported rates (7–13%) and significantly lower than early estimates of 30–60%. This may be related to: (1) Strict control of pressure increase rate; (2) Routine teaching of self-pressure regulation techniques before entering the chamber.

### 4.7 Research limitations and future prospects

Although this study provides preliminary evidence for the application of HBOT in NMOSD treatment, there are still some limitations. First, it is critical to note that the observed ‘de-risking' effect is based on HBOT being used as an adjunctive therapy to stable background immunosuppression. Therefore, the reduction in inflammatory burden should be interpreted as the combined effect of the entire treatment regimen. The specific contribution of HBOT should be further validated in future studies, such as randomized controlled trials with a factorial design, which are specifically designed to disentangle the effects of HBOT from those of baseline immunotherapy. This study is a single-center retrospective cohort study with only 36 cases. Although it meets the minimum sample size required by prior power analysis and provides medium to large effect sizes for primary outcomes, it is still difficult to detect rare adverse reactions and medium to small effects, and the confidence intervals are relatively wide, suggesting uncertainty about the true effect size. The consistency of monocentric diagnosis and treatment processes improves internal validity but limits generalizability to different populations and medical environments. Meanwhile, retrospective design struggles to fully control unmeasurable confounders. Although ANCOVA was conducted to adjust for baseline differences in NLR, PLR, lymphocyte count, and prednisone dosage, the possibility of residual confounding cannot be completely excluded. Immunosuppressive therapies such as AZA, MMF, and RTX, commonly used in the treatment of NMOSD, may influence the neutrophil-to-lymphocyte ratio (NLR) and platelet-to-lymphocyte ratio (PLR). This effect should be considered when interpreting the results, as these treatments could contribute to the observed changes in these markers. To validate the findings of this study, further confirmation is needed through a larger sample size, multi-center, prospective randomized controlled design, particularly focusing on the efficacy differences among different racial groups and populations with varying baseline inflammatory loads.

Secondly, although the improvement of inflammatory markers (such as NLR, PLR) in this study provides evidence for the immunomodulatory effects of HBOT, it did not involve endpoint indicators such as recurrence rate and clinical function. However, this study only provides short-term hematological evidence, and the potential long-term clinical benefits of HBOT in NMOSD require further investigation. Future studies should further evaluate the overall efficacy of HBOT in combination with clinical outcomes (such as EDSS score, recurrence rate, etc.).

Third, rituximab primarily depletes B lymphocytes, whereas our study measured the total lymphocyte count rather than specific lymphocyte subpopulations. Although all patients were in a stable phase and the total lymphocyte count did not show a significant decrease, this remains a limitation as lymphocyte subset changes were not assessed.

In addition to clinical limitations such as sample size, there are still the following gaps at the mechanistic level: Direct evidence gap: There is currently no systematic validation of “HBOT + AQP4-IgG passive transfer model” on the complement-cytokine-neurological damage chain. Key endpoint assessment: It is necessary to correlate the dynamics of CSF/serum sC5b-9, IL-6, and IL-17 with structural indicators such as optical coherence tomography-retinal nerve fiber layer thickness and spinal cord DTI. Dose-response curve: Is the “neuro version” protocol of 2.0 ATA × 105min × 12 sessions optimal? Comparison should be made with 1.5 ATA (low pressure, high frequency) or 2.4 ATA (high pressure, low frequency). Molecular Mechanism Exploration: Transcriptome/Metabolome Joint Analysis of CRISPR-Cas9 Knockout Mice for C3 and IL-6 Receptors, Deciphering the Cross-regulation of Oxygen Sensing-Immune Signals under HBOT.

The EDSS score showed no significant changes. Considering the short observation period of only 4 weeks, axonal remyelination and functional remodeling require months to years; In studies of multiple sclerosis and other conditions, a common pattern is observed where biomarkers change before functional impairments manifest; EDSS is insensitive to early sensory/visual functional fluctuations, and mild visual or sensory improvements are difficult to reflect in the 0–4 score range; Follow-up examinations of Sloan vision and BICAMS may be considered; NMOSD recovery typically follows a “biphasic” curve. After acute injury, inflammation control is required first, followed by the chronic repair phase; The decrease in inflammation markers corresponds to the first phase. Although this study did not observe a significant improvement in EDSS at the 4-week assessment, the rapid and significant decrease in NLR and PLR in the HBOT group suggests potential long-term prognostic benefits. This pattern, where biomarker improvement precedes measurable functional recovery, is well-recognized in neuroinflammatory diseases. Extensive longitudinal evidence in NMOSD specifically shows that elevated NLR and PLR are associated with increased relapse risk and disability progression over 6–12 months, while their decline correlates with functional stability or improvement (22, 24, 38). Therefore, the reduction in these inflammatory markers may serve as a ‘leading indicator' of HBOT's biological effect, reflecting an early optimization of the systemic immune microenvironment. In contrast, functional recovery (as measured by EDSS) likely lags and would require a longer follow-up period to become apparent.

## 5 Conclusion

This study found that hyperbaric oxygen therapy (HBOT) can significantly reduce NLR and PLR levels and increase lymphocyte counts in NMOSD patients by regulating immune cell balance, potentially contributing to immune modulation. In conclusion, the addition of HBOT to standard therapy was associated with more significant immunomodulatory effects in patients with higher baseline inflammatory load (NLR ≥ 3), underscoring its potential as an adjuvant therapy for NMOSD. Although no significant short-term improvement in EDSS was observed within the 4-week study period, the rapid amelioration of systemic inflammation suggests a potential for subsequent clinical functional recovery. Future studies should investigate the long-term efficacy and mechanisms of HBOT and evaluate its therapeutic effects in combination with clinical functional indicators.

## Data Availability

The original contributions presented in the study are included in the article/supplementary material, further inquiries can be directed to the corresponding author.
